# Random Thoughts

**Published:** 1875-10

**Authors:** 


### RANDOM THOUGHTS.

BY NEMO.

The most casual thinker and reader of dental literature can not avoid being impressed with the unsettled state of the therapeutic principles of dentistry and the conflicting ideas of our less informed men as to the most desirable mode of procedure and treatment of pathological conditions of the mouth. And noting the longings of many for a different state of things, will unavoidably come to the conclusion that there is a higher plane for all the votaries of dentistry. And it will never be attained by legislative enactments or traducing others and puffing ourselves. The only way to throttle quackery and elevate dentistry, is for every progressive dentist to make himself superior, scientifically and ethically, to the groveling ones. "Let deeds not words be the rule."

The coming dentist will have to be more conversant with literature and science (especially medical) than is required at the present day to entitle him to the honors of a properly organized dental college. An infinitesimal knowledge of anatomy, physiology, chemistry, pathology and therapeutics will not satisfy an intelligent and discerning public.

A grave delusion has taken hold of the minds of many who have received the degree D. D. S., or some other pseudo-dental title that they are par excellence above all others. Such dupes are cursed with the leprosy.

The model dentist must have a vocabulary, larger than sufficient to mystify an ignoramus, he must eschew all such bombast—as polarization of the brain—by spiritual illumination to differentiate between male and female caries—or ignore by one sweep the rich legacy of thought and research handed down to us by great and good men of the past.

"And, like a peacock sweep along his tail" (pig tail?) Such sayings and doings mark the charlatan.

Our periodical dental literature is susceptible of improvement, much of it is very flippant and nonsensical, for instance the declaration that the saving of a tooth is a greater cause for exultation than the saving of an eye, the information that we have in this country a dentist descended from a titled English family, How we apples swim, etc.

How few dentists comparatively, when business or social intercourse throw them into the company of an educated M. D., and the conversation turns upon medical topics, but feel like pigmies. This is wrong and a tacit acknowledgment of a superiority of knowledge of those subjects with which every dentist should be familiar technically and theoretically. He should by his mental acquirements and gentlemanly deportment command the unqualified recognition of the medical profession as a specialist.

A word especially to the younger dentists who are dissatisfied with their present attainments. Mark out a course of study which will be a life's work, be decided, allow no blandishments to sever you from your course, bearing in mind, "Pluck is a hero, luck is a fool." Four books are indispensable in the work, viz: Webster's Dictionary, unabridged illustrated edition, Dunglison's Medical Dictionary, revised edition, the Bible and Shakespeare, other books and minor details if you possess any will-power will suggest themselves. If the course hinted at is pursued earnestly and perseveringly for one year, you will be enabled to say. "Labor itself is pleasure."

"Be silent, always, when you doubt your sense
And speak, though sure, with seeming diffidence."

